# Neutrophil Activation Status in Stable Coronary Artery Disease

**DOI:** 10.1371/journal.pone.0001056

**Published:** 2007-10-24

**Authors:** Eva Särndahl, Ida Bergström, Veronika Patcha Brodin, Johnny Nijm, Helen Lundqvist Setterud, Lena Jonasson

**Affiliations:** 1 Division of Medical Microbiology, Department of Clinical and Experimental Medicine, Faculty of Health Sciences, Linköping University, Linköping, Sweden; 2 Division of Cell Biology, Department of Clinical and Experimental Medicine, Faculty of Health Sciences, Linköping University, Linköping, Sweden; 3 Division of Cardiology, Department of Medicine and Health, Faculty of Health Sciences, Linköping University, Linköping, Sweden; The Rockefeller University, United States of America

## Abstract

**Background:**

During the last years, neutrophils have emerged as important players in atherogenesis. They are highly activated in peripheral blood of patients with unstable angina. Moreover, a primed state of circulating neutrophils has been proposed in patients with stable angina. Our aim was to investigate the neutrophil activation status in patients with stable coronary artery disease (CAD) at conventional drug treatment.

**Methodology and Principal Findings:**

Thirty patients with stable CAD and 30 healthy controls were included using a paired design. The neutrophil expression of CD18 and high-affinity state of CD11b was analysed by flow cytometry before and after stimulation with chemoattractants. Also, the production of reactive oxygen species (ROS) was determined by chemiluminescence. During basal conditions, the neutrophil expression of CD18 or high-affinity state of CD11b did not differ between patients and controls. Chemoattractants (Interleukin-8 and Leukotriene B_4_) did not increase either the expression or the amount of high-affinity CD11b/CD18-integrins in CAD patients compared to controls, and had no effect on the production of ROS. On the other hand, the ROS production in response to C3bi-opsonised yeast particles and the neutrophils' inherent capacity to produce ROS were both significantly decreased in patients.

**Conclusion/Significance:**

We could not find any evidence that neutrophils in patients with stable CAD were primed, *i.e.* more prone to activation, compared to cells from healthy controls. According to our data, the circulating neutrophils in CAD patients rather showed an impaired activation status. It remains to be elucidated whether the neutrophil dysfunction in CAD is mainly a marker of chronic disease, an atherogenic factor or a consequence of the drug treatment.

## Introduction

During the last decade, new knowledge on neutrophils as active participants in the inflammatory process of atherosclerosis has emerged. Systemic inflammation, involving activated neutrophils, is clearly associated with unstable conditions of CAD [1]–[4]. Moreover, increased numbers of circulating neutrophils is a well known risk indicator of future cardiovascular outcomes, regardless of disease status (reviewed in:[5]). However, the neutrophil activation status in patients with stable CAD has received less consideration and data have so far been inconsistent. Whereas some studies give no evidence for an on-going *in vivo* neutrophil activation [6], [7], others have shown that neutrophils in patients with established CAD or in individuals at high risk for vascular events possess a “primed” character, *i.e.* an increased functional potential *ex vivo* compared to neutrophils from healthy individuals [6], [8]–[10]. In addition, the accumulation of neutrophil-platelet complexes, a possible indicator of neutrophil activation, has been demonstrated in peripheral blood of CAD patients with long-term stable symptoms [7], [11].

The early phase of atherosclerosis involves the recruitment of inflammatory cells from the circulation to the arterial wall [12], [13]. This process is predominantly mediated by cellular adhesion molecules, expressed on the vascular endothelium and on circulating leukocytes. Two of the most important adhesion molecules, expressed exclusively by leukocytes, are CD11a/CD18 (LFA-1) and CD11b/CD18 (Mac-1 or complement receptor 3), which belong to the β2-integrin family [14]. Neutrophils in patients with acute coronary symptoms display increased number of β2-integrins compared to patients with stable CAD [7], [11], while no up-regulation of CD11b/CD18 has been found on neutrophils from patients with stable CAD compared to healthy controls [7], [10], [11]. However, the absolute number of integrins on the cell surface does not necessarily reflect their functional role. To obtain binding of neutrophils to the vascular endothelium, β2-integrins must be turned into an active state; a process accomplished via receptors for chemokines and chemotactic factors by a so called inside-out signalling [15]. Integrins have been suggested to increase their adhesive properties through inside-out signalling by two distinct mechanisms [16]; the first being a change in affinity for the ligand, and the second involving changes in lateral surface motility and clustering of integrins. An increase in β2-integrin affinity is achieved by conformational changes of the integrin, thereby exposing an epitope that enables ligand binding, the so called I-domain [17].

In the present study, we hypothesized that neutrophils in patients with stable CAD were in a primed or pre-activated state compared to neutrophils in healthy individuals. The neutrophil activation status, involving the expression, affinity state and signalling capacity of β2-integrins during basal and stimulatory conditions as well as the innate ROS production, was investigated in a paired patient-control design.

## Results

### Clinical and laboratory characteristics of subjects

Clinical and laboratory characteristics of patients and controls are given in Table 1. The use of medication differed significantly between the two groups. A small number of controls received treatment with low-dose aspirin, beta-blockers, angiotensin-converting enzyme inhibitors or statins due to the presence of risk factors, such as hypertension and hyperlipidemia. There were no differences in the amount of C-reactive protein (CRP) or leukocyte blood count between patients and controls.

**Table 1:** Clinical and laboratory characteristics of patients and controls.

	Patients	Controls	*p* values
Total no.	30	30	ns
Female/Male (no.)	5/25	5/25	ns
Age (years)	63 (59–67) 1)	63 (60–68)	ns
Smokers n (%)	4 (13)	4 (13)	ns
Blood pressure (mm Hg)			
Systolic	135 (120–146)	141 (130–152)	ns
Diastolic	83 (78–91)	84 (78–92)	ns
Body mass index (kg/m^2^)	27 (24–29)	25 (24–29)	ns
Medication n (%)			
Beta blockers	28 (93)	3 (10)	<0.001
ACE-I/ARB 2)	16 (52)	2 (7)	<0.001
Statin	30 (100)	3 (10)	<0.001
Low-dose Aspirin	30 (100)	1 (3)	<0.001
Laboratory variables 3)			
CRP 4) (mg/mL)	0.9 (0.3–1.8)	1.2 (0.4–2.2)	ns
Blood glucose (mmol/l)	5.1 (4.4–5.9)	5.8 (5.2–6.5)	<0.01
Lipids (mmol/l)			
Total cholesterol	4.5 (4.1–5.0)	5.2 (5.0–6.0)	<0.01
Low-density lipoprotein (LDL) cholesterol	2.2 (1.9–2.8)	2.9 (2.5–3.4)	<0.05
High-density lipoprotein (HDL) cholesterol	1.4 (1.2–1.6)	1.5 (1.3–2.1)	ns
Triglycerides	1.5 (1.0–1.9)	1.2 (0.9–1.8)	ns
Blood count (cells/µL)			
Leukocytes	6.1 (5.3–6.8)	5.6 (4.8–6.7)	ns
Neutrophils	3.0 (2.6–3.5)	2.7 (2.3–3.9)	ns

1) Data are given as median (inter-quartile range).

2) ACE-I, angiotensin converting enzyme inhibitor; ARB, angiotensin receptor blockade

3) Measured at the accredited clinical chemistry laboratory at Linköping University Hospital, Sweden.

4) CRP, C-reactive protein

### Baseline expression and high-affinity state of β_2_-integrins on neutrophils

**Figure 1:**
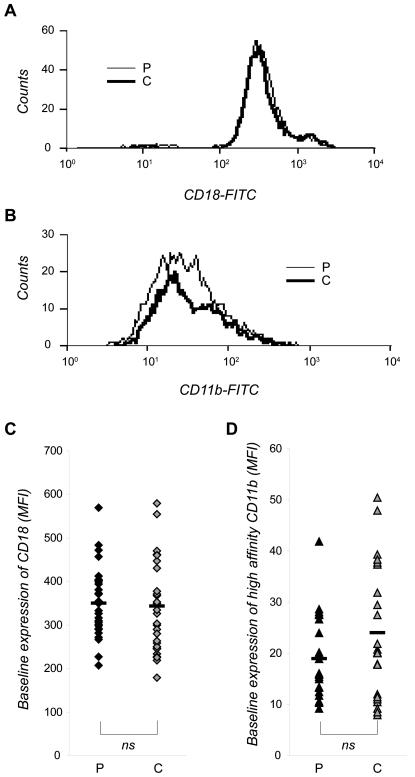
Baseline expression of CD18 and high-affinity state CD11b on neutrophils in patients with stable CAD. Whole blood from patients (P) and individually matched control persons (C) were incubated at 37°C with (A,C) the MHM23-antibody or with (B,D) the CBRM1/5-antibody for 5 and 10 min, respectively, whereafter the samples were incubated on ice. Contaminating erythrocytes were removed by lysis, and antibody bound to CD18 or to CD11b-integrins displaying the high-affinity epitope was detected by FACS analysis. The cells were gated to identify the neutrophil population, and 10 000 cells were analyzed in each sample. Data are shown as one representative histogram plot (A,B) or as median of the mean fluorescence intensity (MFI) values (C,D) from 30 (A,C) and 25 (B,D) experiments per cohort done in triplicate. Mean value is indicated as a black hyphen. Wilcoxon signed-ranks test was used to determine significance between each patient and its individually matched control.

Using flow cytometry, the mean fluorescence intensity (MFI) values revealed that patients with stable CAD and control subjects expressed the same amount of β2-integrins on their neutrophils (349±80 and 337±104, respectively; mean MFI±SD; Fig. 1A,C). Also, no differences in the amount of β_2_-integrins exposing the high-affinity epitope could be detected when comparing neutrophils from patients and controls (19±8 and 24±12, respectively; Fig. 1B,D).

### β2-integrin up-regulation on neutrophils upon IL-8 or LTB_4_ stimulation

**Figure 2:**
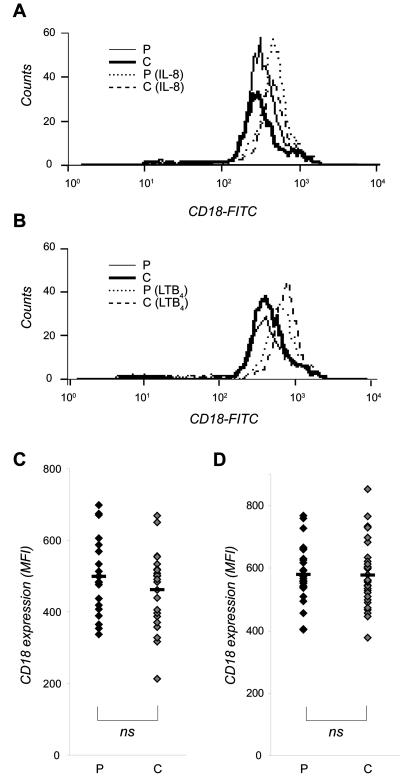
IL-8 and LTB_4_ induced expression of CD18 on neutrophils from patients with stable CAD. Whole blood from patients (P) and individually matched control persons (C) were stimulated for 10 min at 37°C with (A,C) IL-8 (10 ng/mL) or (B,D) LTB_4_ (100 nM), and the samples were co-incubated with the MHM23-antibody during the last 5 min of stimulation. The stimulation was stopped by incubation the samples on ice, and contaminating erythrocytes were removed by lysis. Antibody bound to CD18 was detected by FACS analysis. The cells were gated to identify the neutrophil population, and 10 000 cells were counted in each sample. Data are shown as one representative histogram plot (A,B) or as median of the mean fluorescence intensity (MFI) values (C,D) from 22 (A,C) and 30 (B,D) experiments per cohort done in triplicate. Mean value is indicated as a black hyphen. Wilcoxon signed-ranks test was used to determine significance between each patient and its individually matched control.

The pro-inflammatory chemoattractants IL-8 and LTB_4_ were used to study up-regulation of β2-integrins on neutrophils. In both patients and healthy individuals, stimulation with IL-8 induced an up-regulation of β2-integrins (148%±30 and 152%±37, respectively; Fig. 2A,C), and an even stronger up-regulation was induced by LTB_4_ (172%±37 and 180%±38, respectively; Fig. 2B,D) compared to unstimulated cells. No significant difference was detected between patients and controls in regard to the total expression of β2-integrins (Fig. 2C and D) or in the increased percentage of up-regulated β2-integrins upon chemoattractant stimulation (data not shown).

### The expression of high-affinity state β2-integrins on neutrophils upon IL-8 or LTB_4_ stimulation

**Figure 3:**
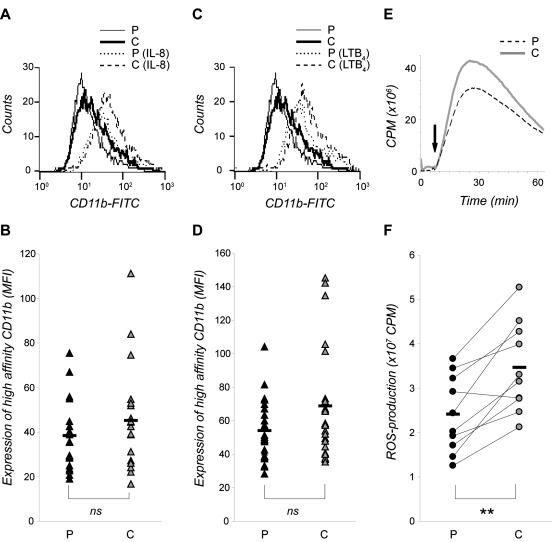
IL-8 and LTB_4_-induced expression of high-affinity state CD11b on neutrophils from patients with stable CAD. (A–D) Whole blood from patients (P) and individually matched control persons (C) were incubated for 1 min with CBRM1/5 at 37°C, before stimulated for 10 min with (A–B) IL-8 (10 ng/mL) or (C–D) LTB_4_ (100 nM) at 37°C. The stimulation was stopped by incubating the samples on ice, and contaminating erythrocytes were removed by lysis. Antibody recognizing integrins displaying the high-affinity epitope was detected by FACS analysis. The cells were gated to identify the neutrophil population, and 10 000 cells were counted in each sample. Data are shown as one representative histogram plot (A,C) or as median of the mean fluorescence intensity (MFI) values (B,D) from 20 (A–B) and 25 (C–D) experiments per cohort done in triplicate. (E–F) Isolated neutrophils from patients and control persons were stimulated with C3bi-opsonized yeast particles (5×10^6^/mL), and the production of ROS was measured continuously for 60 min at 37°C. Data are expressed as counts per min (cpm) and shown as one representative histogram plot (E) or as total ROS production peak value from 10 experiments per cohort (F). Mean value is indicated as a black hyphen. Wilcoxon signed-ranks test was used to determine significance between each patient and its individually matched control (** represents significant difference; *p*<0.01).

The inside-out regulation of the high-affinity β2-integrins was evaluated in neutrophils stimulated with IL-8 or LTB_4_. Stimulation with IL-8 (Fig. 3A–B) and LTB_4_ (Fig. 3C–D) induced a prominent increase in the amount of CD11b exposing the high-affinity epitope compared to unstimulated neutrophils in both patients and controls (230%±81 and 225%±76; 308%±102 and 316%±95, respectively). No significant difference in the total number of high-affinity β2-integrins (Fig. 3B and D) or in the increased percentage of high-affinity β2-integrins (data not shown) was detected between neutrophils from patients and controls upon chemoattractant stimulation

### β2-integrin signalling capacity in neutrophils

The signalling capacity of β2-integrins on neutrophils was determined by measuring ROS production in response to C3bi-opsonized yeast particles. We found that neutrophils from the patients produced significantly lower levels of ROS compared to their individually matched controls (with a mean reduction of 30%±17; Fig. 3E–F). In support of previous studies on neutrophils from healthy blood donors [18], the production of ROS elicited by C3bi-opsonized yeast particles in neutrophils isolated from patients was mainly intracellular (data not shown).

### ROS production in neutrophils upon IL-8 or LTB_4_ stimulation

**Figure 4:**
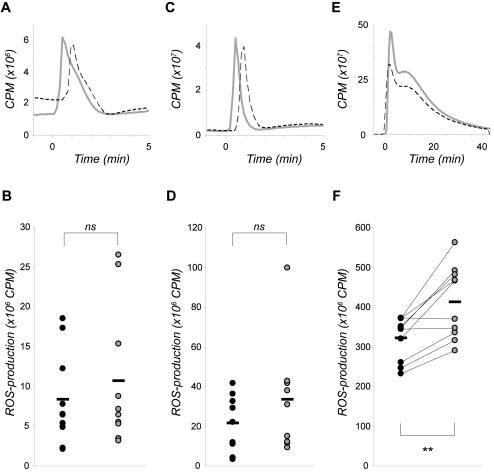
IL-8 and LTB_4_ induced ROS production in neutrophils from patients with stable CAD. The production of ROS in response to (A–B) IL-8 (100 ng/mL), (C–D) LTB_4_ (100 nM), or (E–F) PMA (50 nM), was measured in isolated neutrophils from patients (black dashed line and black dot, respectively) and individually matched controls (grey line and grey dot, respectively). Data are expressed as counts per min (cpm) and shown as one representative histogram plot (A,C,E) or as total ROS production peak value from 9–10 experiments per cohort (B,D,F). Mean value is indicated as a black hyphen. Wilcoxon signed-ranks test was used to determine significance between each patient and its individually matched control (** represents significant difference; *p*<0.01).

To investigate if neutrophils from patients with stable CAD displayed a more primed character, the total ROS production (*i.e.* intracellularly produced plus extracellularly released) was measured in response to IL-8 and LTB_4_. Both IL-8 and LTB_4_ elicited mainly extracellular ROS production (data not shown). The ROS responses were of the same magnitude in neutrophils from patients as in neutrophils from controls (Fig. 4A–D). However, PMA (*i.e.* a non-receptor mediated response) elicited significantly lower production of ROS in neutrophils from patients compared to neutrophils from individually matched controls, with a mean reduction of 21%±12 (Fig. 4E–F).

## Discussion

The present study investigated the activation status of neutrophils in patients with stable CAD by determining the cells adhesive property and their ability to produce ROS. We found that the baseline expression of β2-integrins did not differ between neutrophils in patients and healthy controls; neither did the amount of β2-integrins exposing the high-affinity epitope. The results are in line with previous reports showing similar levels of β2-integrins on neutrophils in patients with stable CAD as in healthy controls [6], [7], [10], [11]. However, the number of integrins on the neutrophil surface does not reflect the functional state of the integrin. In order to enable cellular adhesion, a conformational change of the β2-integrin is needed to increase its affinity [19]. Our results, demonstrating similar levels of high-affinity CD11b-integrin in patients with stable CAD as in healthy controls, provide new knowledge and extend thereby data from previous reports [6], [7]; also contradicting an *in vivo* neutrophil activation in stable CAD.

IL-8 and LTB_4_ are both potent leukocyte chemoattractants, known to promote several inflammatory disorders, including atherosclerosis. Serum levels of IL-8 are elevated in patients with stable CAD [20], and in healthy individuals elevated levels of IL-8 are associated with an increased risk of future CAD [21]. As expected, the expression and affinity of β2-integrins, as well as the ROS production were increased in neutrophils upon stimulation with IL-8 or LTB_4_. However, these stimuli-induced responses of neutrophils in patients were comparable to the ones detected in healthy individuals. Data from previous studies have suggested that neutrophils in patients with stable CAD or in individuals at high risk for vascular events possess a primed character compared to neutrophils from healthy individuals, *i.e.* the cells are “hyperactive” *ex vivo* as assessed by increased chemotactic activity and increased production of ROS, LTB_4_ and IL-8 [6], [8], [9]. However, our data could not support the hypothesis that neutrophils in patients with stable CAD are more prone to activation.

In a recently published study, a different approach was used to study neutrophil activation. In a small group of CAD patients, a skin blister model was applied in order to describe transmigration of neutrophils at different stages of interstitial inflammation [10]. *In vivo* transmigrated neutrophils in patients showed an increased capacity to release gelatine-associated lipocalin and matrix metalloproteinase-9 in a milieu of intermediate, but not intense, inflammation. The authors thereby proposed that the dysfunctional neutrophil response in CAD patients was a consequence of a primed state of circulating neutrophils. It is possible that the stimulation with IL-8 and LTB_4_, used in our study, represented a too intense inflammatory state in which a primed character of the neutrophils was abolished by *e.g.* receptor desensitization [22], [23]. Arguing against this is that IL-8 and LTB_4_ are intermediate chemoattractants, known to induce more moderate cellular responses [24]. In addition, it could be argued that the stimulation with IL-8 and LTB_4_ is a more clinically relevant model with regard to atherosclerosis than the skin blister model [10].

An aberrant neutrophil activation status in atherosclerosis, not only involving priming but also refractivity to further stimulation, has been discussed. Paulsson and co-workers [10] found that *in vivo* transmigrated neutrophils in CAD patients had a reduced capacity to up-regulate CD11b and to produce H_2_O_2_ compared to neutrophils in healthy controls. In the present study, we show that neutrophils in patients with stable CAD possessed a functional inside-out regulation in relation to β2-integrin expression and affinity. However, upon stimulation with C3bi-opsonised yeast, which binds and activates CD11b/CD18 [25], the β2-integrin signalling was significantly decreased in neutrophils from CAD patients compared to controls. This reduced response to C3bi-opsonised yeast might also reflect the adhesive property of the β2-integrin [26], but regardless of whether the observed down-regulated signalling capacity was due to a dysfunctional signalling capacity *per se* or resulted from decreased adhesion, the results indicate that inside-out signalling regulating CD11b/CD18-integrin avidity was down-regulated in patients with stable CAD. In addition, the non-receptor-mediated ROS production by PMA was significantly decreased in patients, suggesting that the optimal capacity to generate ROS was deficient in patients. Altogether, our data point towards a dysfunctional activation status of neutrophils in CAD patients, involving cellular processes mediated by β2-integrins.

In contrast to our previous data [11], the CAD patients in the present study were without signs of systemic inflammatory activity, as assessed by CRP and neutrophil counts. One plausible explanation is that each patient in the present study was treated with both low-dose aspirin and statin. Aspirin and statin are both well known CRP-lowering agents (reviewed in: [27]). A primed neutrophil response has also been shown to attenuate upon therapy with these drugs. By affecting protein prenylation, statin modulates a variety of signalling molecules; many of which are involved in regulating adhesion (reviewed in: [28]). In high-risk individuals, a 4-week treatment with statin was able to reduce the synthesis of IL-8 in neutrophils and decrease the neutrophil migration and chemotaxis [9]. Aspirin influence a number of mechanisms and signalling pathways involved in neutrophil activation, for instance attenuating the expression of IL-8 [29] and the LTB_4_-induced migration of neutrophils [8]. Aspirin-triggered lipoxins attenuates the expression of β2-integrins [30], decreases the LTB_4_-induced adhesion-state of neutrophils by affecting the avidity of the β2-integrin [31], and inhibits neutrophil adhesion to activated endothelial cells [32].

To conclude, the present study does not give any support for a role of neutrophil priming in stable CAD. The neutrophils in patients with stable CAD were not more activated *in vivo* than were cells in healthy controls, neither were the neutrophils in patients more prone to activation *ex vivo*. Rather, the neutrophils in patients displayed a decreased β2-integrin signalling and/or adhesion capacity and a deficient intrinsic capacity to generate ROS. Although the results indicate that the drug therapy of today is efficient in maintaining the neutrophils in a non-adhesive manner; an outcome that most certainly has a positive impact on the prognosis, the clinical relevance of neutrophil dysfunction in CAD remains to be elucidated. Is it mainly a marker of chronic inflammatory disease or a factor with impact on the progress of CAD?

## Materials and Methods

### Subjects

Thirty patients with angiographically verified stable CAD were recruited from the Department of Cardiology, Heart Centre, Linköping University Hospital, Sweden. The patients had effort-related angina in accordance with the Canadian Cardiovascular Society functional classes I and II without any worsening of symptoms the latest 6 months. Patients were excluded if they were>65 years old, had severe heart failure, immunologic disorders, neoplasm disease, evidence of acute or recent (<2 months) infection, recent major trauma, surgery or revascularization procedure, or treatment with immunosuppressive or anti-inflammatory agents (except low-dose aspirin). Each patient was matched, regarding age and sex, with a clinically healthy control randomly selected from a population register representing the hospital recruitment area. The control subjects were anamnestically healthy and with normal routine laboratory tests. All patients and control persons gave informed oral consent. The appointed ethics committee at Linköping University approved the research protocol, and the study was conducted in accordance with the ethical guidelines of Declaration of Helsinki.

### Collection of blood

For each experimental set-up, venous peripheral blood was drawn from one patient and its matched control in the morning after a 12 h fast, and collected in heparin-containing vacutainer tubes. The samples were blinded during all experiments.

### Flow cytometry

**Figure 5:**
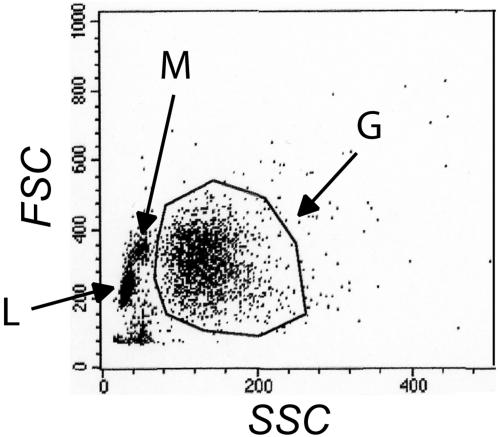
A representative gating to identify the neutrophil population in the flow cytometry analysis. G: Granulocytes, M: Monocytes, L: Lymphocytes, FSC: Forward scatter, SSC: Side scatter.

Immuno-labelling was performed, as previously described [31], to investigate changes in the total expression of β2-integrin and in the expression of β2-integrins displaying the high-affinity epitope. In short, whole blood (50 µl/sample) was pre-heated at 37°C. In order to study the high-affinity state of the β2-integrin, a monoclonal FITC-conjugated mouse anti-human CD11b antibody recognizing the ligand binding domain (*i.e.*, the I-domain [33]; CBRM1/5; Biosite) was used. This antibody was given 1 min before stimulation with Interleukin-8 (IL-8; Sigma-Aldrich) or and Leukotriene B_4 _(LTB_4_; Larodan Fine Chemicals AB). In samples used for detecting the plasma membrane expression of CD18, the monoclonal FITC-conjugated mouse anti-human CD18 antibody (MHM23; DakoCytomation) was added 5 min after stimulation. For all samples, the stimulation was stopped by incubating the samples on ice. Contaminating erythrocytes were removed by ice-cold cell lysis (NH_4_Cl 150 mM, KHCO_3_ 10 mM, EDTA 100 µM) for 5 min at 15°C. The cells were collected and fixed in ice-cold 0.1% paraformaldehyde (PFA). Mean fluorescence values from 10 000 cells/sample were determined by flow cytometry using a Becton Dickinson FACSCalibur (Becton Dickinson, San Jose, CA/USA). Cell populations were identified by plotting FSC versus SSC excluding cell debris, followed by gating and further analysis of the neutrophil (granulocyte) population (Fig. 5). Auto-fluorescence of unstained cells was routinely analyzed. Unspecific binding was determined using a FITC-conjugated isotypic mouse anti-human IgG1 (DakoCytomation).

### Preparation of neutrophils

Neutrophils were prepared by density gradient centrifugation, as previously described [31]. In short, freshly drawn heparinized blood was layered on top of Lymphoprep™ and Polymorphprep™ (Axis-Shield AS), and centrifuged in a swing-out centrifuge (400×g, 30 min, RT). The granulocyte band was collected, washed and resuspended in a small volume of PBS (pH 7.3; made in-house), and concentrated by a short spin. Contaminating erythrocytes were removed by hypotonic lysis, and the cells were washed, resuspended in Krebs-Ringer glucose buffer (KRG; 10 mM glucose, 1 mM Ca^2+^, and 1.2 mM Mg^2+^; pH 7.3; made in-house) without Ca^2+^, and kept on ice.

### Opsonization of yeast particles

Serum opsonized yeast particles were prepared, as previously described [34]. In short, heat killed yeast (*Saccharomyces cerevisiae*) were opsonized with 25% normal human serum (AB^−^) for 30 min at 37°C. The yeast particles were resuspended in KRG, homogenized, and adjusted to a concentration to 5×10^7^/mL, before added as stimuli at ratio 5∶1 (yeast/cell).

### Determination of ROS production

Chemiluminescence was used to determine the ROS production of isolated neutrophils from 10 patients with stable CAD and 10 control subjects, respectively. The measurements were performed, as previously described [35], using a six-channel Biolumat LB9505 (Berthold Co., Wildbad, Germany). Neutrophils (5×10^6^/mL) were diluted in KRG in polypropylene tubes to a total volume of 1 mL (after addition of the stimulus). Total ROS production was measured using luminol (5-amino-2,3-dihydro-1,4-phthalazinedione; 56 µM; Sigma), and horseradish peroxidase (HRP; 4 U/mL; Roche). For measurements of the intracellular ROS production samples contained luminol (56 µM), superoxide dismutase (SOD; 50 U/mL; Roche) and catalase (2000 U/mL; Roche). Extracellular ROS production was measured using isoluminol (6-amino-2,3-dihydro-1,4-phthalazinedione; 56 µM; Sigma) and HRP (4 U/mL). The samples were pre-heated for 5 min before addition of stimuli, and the temperature was kept at 37°C during the measurements. Light emission, reflecting ROS interacting with luminol/isoluminol was continuously monitored. The peak value was registered for each sample, and expressed as counts per minute (cpm). PMA (Phorbol 12-myristate 13-acetate; Sigma), which directly activate protein kinase C, was used to examine the maximal ROS production elicited in the cells.

### Statistics

Statistics were performed using the SPSS program (version 11.5). For all clinical and laboratory characteristics, non-parametric tests were used since data, in general, were non-normally distributed. These data were presented as median (inter-quartile range). The significance of any difference between patients and controls was tested by using Mann-Whitney U-test. Data on β2-integrin state and ROS production were analysed by Wilcoxon signed rank test to compare levels between a patient and its paired control. A *p*<0.05 was considered significant.
